# Supplementary material for: Soil-feeding termites build islands of soil physical and chemical fertility in pastures in Colombian Amazon
Source: PLoS One. 2025 Apr 29;20(4):e0322186. doi: 10.1371/journal.pone.0322186 (PMC12040218; doi:10.1371/journal.pone.0322186)
Supplement: Attachment — Submitted filename: 20240709_Review.docx [file pone.0322186.s001.docx]

**Summary of the research**

The study was conducted in three pastures located in the northwestern Colombian Amazon, characterized by a mean annual precipitation of about 3700 mm. The soils are leached, nutrient deficient, acidic, and compacted red Ultisols with aluminum concentrations potentially toxic to plant growth. The study addresses the important topic of soil-organism interaction.

The authors investigated how physicochemical properties of epigeal mounds built by the soil-feeding termite species *Patawatermes turricola* differ from termite-unmodified reference soil (habitat topsoil). Physical soil parameters assessed included aggregate stability, macro- and microporosity, and bulk density. Chemical parameters included soil organic carbon, exchangeable acidity, aluminum toxicity, cation exchange capacity, base saturation, and concentrations of macronutrients (plant-available phosphorus, Mg, Ca, K, Na). These parameters are indicators of soil health, soil fertility, toxicity, soil structure, that is parameters crucial for plant establishment and development.

Previous studies mostly investigated physicochemical parameters of epigeal mounds built by fungus-growing termites in semi-arid agroecosystems and savannas in Africa, where fungus-growing termites are the main year-round active decomposers and soil bioturbators. In contrast, unfortunately, there is limited knowledge about the characteristics of epigeal mounds built by neotropical soil-feeding termites, and their role as ecosystem engineers in agroecosystems of the New World tropics.

The topic addressed in the article clearly lies in the scope of the journal PLOS One. The sample size of 54 mound soil and control soil samples is sufficient (and the methods used are appropriate) to draw robust conclusions about the characteristics of *P. turricola* mounds in the study sites.

However, I found some important information lacking, such as how the *P. turricola* mounds could improve soil health, alongside details on mound densities, mound soil quantities, turnover/erosion rate. It would be beneficial to explain why *P. turricola* mounds and the analyzed physicochemical parameters were selected. Additionally, it would be informative to know if there are other epigeal-mound builders in the region, whether soil-feeding termites are the primary decomposers/ bioturbators, and the presence and impact of other soil macrofauna, particular earthworms, given the region's high annual precipitation.

These points should be addressed to strengthen the manuscript and provide a comprehensive understanding of the study's findings and implications. Most of this information should be readily available. If data on mound soil quantity are lacking, this could be acknowledged and discussed without impacting the manuscript's acceptance. I suggest a thorough revision of the Discussion section, ensuring the terms used (e.g., macro- and microporosity) align with their definitions in the cited literature (see comments below).
Additionally, the manuscript would benefit from English language editing.

Overall, the article is suitable for publication pending revisions addressing both minor and major points. Specific comments and questions for the authors are provided below, unfortunately not differentiated into major and minor points - for which I apologize.

**Soil-feeding termites build islands of soil physical and chemical fertility in pastures in Colombian Amazon**

**Remark:** Abbreviate the genus after first introduction when referring to termite species (*P. turricola*)

**Abstract:
Line 20:** … feeding and nesting activities and the construction of biogenic structures …

**E.g. Line 21ff:** State early in the abstract that the mounds were built by soil-feeding termite species. For example, "mounds constructed by the soil-feeding termite species *Patawatermes turricola*…” (Why this species? Is *P. turricola* the predominant termite species/most conspicuous, most widespread mound-builde/macrofauna?

… some useful information about study site would be habitat type (pasture), region (northwestern Colombian Amazon), mean annual precipitation about 3800 mm, - soil type Ultisol, characterized by - leached, nutrient deficient, .. (most prominent parameters potentially influenced by soil-feeding termites).

Briefly state your hypothesis and, in the last sentence, relate the findings to your hypothesis.

Why did you select/study these physicochemical characteristics of these mounds? (Example: due to trampling of animals, the pastures have especially compacted soils? Therefore aggregate stability and macro/microporosity of mound soil?

Define " better values/improvement" in your context: beneficial for plant growth, other macrofauna, soil structure? How can mound properties positively influence the habitat? Via erosion of mound-soil, turn-over? As stated by Sarcinelli et al. (2013) “With time and abandonment, termite mounds are eroded, and their material is redistributed on the soil surface, potentially creating a soil environment more favorable to plant establishment and development.”

How can findings be useful for habitat management or conservation?

**Keywords**: “bioturbation” – Soil bioturbation is not directly addressed or quantified in the study.
Information about *P. turricola* mounds in the manuscript: 30 cm high – Was the basal diameter/circumference assessed? Can you roughly estimate the amount of soil bioturbated to construct these mounds? This could contribute information on bioturbation during mound construction.

**INTRODUCTION**

**Lines 47ff:** The authors describe soil-feeding termites as feeding group. It would be good to very briefly name which other feeding groups do exist (or are important in the study region). For instance referring to the classification of Donovan – the authors last reference [62]: (Donovan *et al.*, 2001)
🡪 as well because of Line 56:… the magnitude of these differences depends on feeding habits, the soil properties, the termite species, mound age, vegetation, and land use..”
… “This fecal building process results in unique physico-chemical properties of these termite mounds, which are characterized by higher moisture and higher concentration of organic matter and cations.”
→ Are these mound parameters characteristic for soil-feeder mounds? Is higher moisture content in mounds also important in region with 3900 mm mean annual rainfall?

Are earthworms important in the study region/sites as well? (termites known to be dominant decomposers and soil bioturbators especially in dry, degraded habitats – as well in your sites?)

**Line 61ff:** “…therefore playing an important role in soil fertility. This is especially true in the Amazon basin, where extensive livestock production has led to the establishment of vast pasture areas (269,000 km² between 1960 and 2019, [15]), which are in some stage of degradation due to the low investment in soil management and nutrient addition”

🡪 What do you expect as most important effects of the selected soil-feeders and their mounds in the study region/study sites?

**Line 66:** “However, despite the high prevalence of epigeous termite mounds in tropical pastures..”
🡪 Is the density of *P. turricola* mounds high?

**Lines 67f**: “…being under-appreciated by farmers who commonly associate high mound density with pasture degradation”

🡪 what do the farmers do? What would you want farmers to appreciate? Are there plans to convert the pastures into farmland? Why is it important to know the mounds physicochemical characteristics?

**Lines 72ff**: “We hypothesized that mound-building activities by soil-feeding termites improve the soil physical and chemical properties by creating island of soil fertility that can be particularly important in those nutrient-poor agroecosystems. To test this hypothesis, we conducted a field study to assess the content of soil macronutrients and soil organic C, the soil acidity, soil bulk density, porosity and aggregation of the termite mounds and their surrounding soils.”

**Questions:** Did your expectations on the effect of soil-feeding termite mounds in the study sites influence the soil parameters selected for your study? Are the selected parameters important for soils present in your area? For example see comment to Lines 86f (aluminum toxicity).
🡪 Short reasoning of parameter selection, e.g. aggregate stability: important physical indicator of soil health, protects organic matter accumulation, improves soil porosity, drainage and water availability for plants, decreases soil compaction, supports biological activity, and nutrient cycling in the soil.

**MATERIALS AND METHODS**

**Study area and sampling design**

**Question:** Do you have photos showing *P. turricola* mounds in your study sites?

**Line 84ff:** “The regional climate is classified as AF type (Köppen classification) with an average annual rainfall of 3793 mm…”
-- The type is “Af” (second letter lowercase) and maybe add meaning i.e. Af=”tropical rainforest climate or equatorial climate”… information regarding rainfall distribution, e.g. …mono-modal rainfall distribution, with maximum precipitation between the months of ?? and ??.

Study period? When (year, months) were soil samples taken?

**Line 86f:** …Ultisols… - Are soluble Al^3+^ concentrations in a range that is toxic to plants? If so, perhaps "Al toxicity" would be more appropriate than "high Al content." Sarcinelli *et al.* (2013) stated in their review that aluminum toxicity was apparently reduced by the mound-building activity of termites. This provides an argument for analyzing available Al^3+^ concentrations in the present study.

**Lines 93ff:**
What are (roughly) the sizes of the three pastures studied and how far are they separated from each other? Did they differ in altitude, topography, site history, or any other characteristics? What were criteria to select these three pastures?
And: In the section Data analysis (GLM, GLMM) it is written that plot was set as a random effect. Does “plot” refer to the three pastures?

Do all epigeal termite mounds in the study sites belong to *P. turricola*? What is the shape of the mounds - conical? In their study, Duran-Bautista *et al.* (2023) assessed epigeal termite mounds in pastures which seem to be pretty close to the sites in the present study. There, a second soil-feeding termite built mounds although in lower densities, and the *P. turricola* mounds seem to be somewhat smaller.

**Line 100: “**A total of 20 transects, each measuring 20x2 meters, were established across the paddocks.” – How did you select the locations and the directions of the 20 belt-transects?
What was the distance between the transects? How many transects placed in each pasture?

**Line 101:** “…54 mounds of *Patawatermes turricola* were carefully assessed.” Which parameters have been assessed? Was the basal area /circumference and height of each mound assessed? Can you estimate the amount of mound soil?

Can you estimate the density of *P. turricola* mounds in your study sites? This information is better comparable with other areas and all the necessary information seem to be available:
20 belt-transects of 20x2m assessed = 800 m² 🡪 54 *P. turricola* mounds in 800 m² 🡪 Density: 6.75 mounds in 100m² or per hectare (or whatever area is appropriate to state) .

**Line 101ff:** Soil samples taken from the mound: monoliths and cylinders at height of 30cm: How did you take these samples in a height of 30 cm from the base if mounds are 30 cm tall? Did you have enough space in between these two undisturbed samples?
Was there a reason to take the habitat soil samples in a distance of 5 m? Why assumed to be unmodified by termite action/ not influenced by mound erosion? “at depth of 10cm” means samples taken in topsoil horizon 0-10cm, right?

**Line 108ff:** Three disturbed soil samples of mound: top, middle, bottom 🡪 from the outer mound wall, right? How much soil did you (roughly) take each to form the composite samples? The disturbed control soil samples taken in a distance of 5 m next to the mound: as well three replicates taken for composite samples of habitat topsoil? What was the distance between these three topsoil samples/selection of soil sampling location?

**General remark:**
I found it quite difficult to refer to the topsoil samples taken in a distance of 5 m next to each mound as "adjacent soil". Another term might be easier - e.g. "(recently) unmodified habitat soil", "unmodified topsoil", "control soil", “adjacent, termite-unmodified control soils”, “termite-unmodified reference soil”... whatever term seems most appropriate to indicate that when compared to the control topsoil of the habitat, the differences in physicochemical parameters are due to the soil modification by termites during mound construction.

**Data analysis**

**Lines 153ff:**
To facilitate citations for the packages used in R (some references are provided), it might be easier to first say that R 4.2.1 was used to carry out all statistical analyses…
Which test was used to assess normal distribution?
For which data Poison Regression and for which Negative Binomial Regression?

For example:

The statistical analyses were conducted using R software version 4.2.1 [reference].
The xxx test was used to assess normal distribution/The normality of residues was verified with the xxx test./ …
A principal component analysis (PCA) was performed with the R add-on package ade4 [reference] to discriminate the different soil types (mound soil, habitat soil) on the basis of the soils’ physicochemical properties../ to determine the physicochemical parameters that best-captured variance within each soil type …. (Any standardization of variables which should be stated?)
Additionally, to test the hypothesis that decisive soil parameters significantly differ between recently unmodified topsoil samples (control soil) and soil modified by soil-feeding termites during nest construction (mound soil), each parameter was individually evaluated. Soil parameters with normal data distribution (soil porosity, bulk density, organic carbon,..) were analyzed using a Generalized Linear Model (GLM) with the glm2-package [Reference].. Soil properties whose data did not follow a normal distribution (cations potassium, calcium,…, macro- and microporosity,…..) were analyzed using a Generalized Linear Mixed Model (GLMM). Negative Binomial Regression used for …. and Poisson Regression for ….
In all models, soil type was set as a fixed effect, and plot as a random effect. Model selection was based on the Akaike information criterion (AIC), Bayesian information criterion (BIC), and overdispersion.
The R package multcomp [reference] was used for Fisher's … Unless otherwise stated, the significance threshold was set to a p-value of 0.05.

ade4-package: (Dray and Dufour, 2007)

glm2-package: (Bates D *et al.*, 2015)

multcomp-package: (Hothorn *et al.*, 2008)

**RESULTS:**

**Suggestion/Question:**
Before elaborating on the variability explained by axes 1 and 2 in the PCA, it might be good to start this section with stating the most important differences found between mound soil and habitat soil (and the reference to their values).

“Mound soil and habitat soil differed remarkably in the analyzed physicochemical properties… most prominent were differences in… “

And since no distinction between physical and chemical parameters was made in Fig 2 /PCA, it could be easier in the Results-section to report physical and chemical parameters in one section/table (e.g. first listing the physical, then the chemical parameters, even subheading in table would be possible). The structure of Table 1 and Table 2 is identical as well...

Or what was the intention to have different subsections and tables for physical parameters and chemical parameters after their joined presentation in Fig 2?

**Remarks to the PCA:**
It seems, that all analyzed soil parameters are shown in Fig 2/PCA. It would be helpful if the loading matrix could be provided/ table with the loadings of the variables on the principal components indicating which variables contribute most to each component. Those parameters which do not contribute significantly should/could be excluded from the figure – if no other considerations for showing them here as well (e.g. needed for interpretations)

Which other parameter can be excluded – for example, macro-, micro-, total porosity – highly correlated/ not all three are needed.

Then describe what each principal component represents based on the loadings of the variables to describe which patterns or trends are visible? PCA analysis indicated that F1 and F2 components explained the maximum variance with respect to different soil parameters (cumulative percentage of variance was 49.3%) … (Fig..)

**Lines 197-199: Caption of Figure 3** “Fig 3. Porosity and bulk density analysis: a) Total: total porosity proportion, Macro: macroporosity, Micro: micro-porosity in AS: Adjacent Soil and M: Mound Soil. b) bulk density in AS: Adjacent Soil and M: Mound soil, vertical bars represent standard error.”

🡪 What do authors mean by “vertical bars represent standard error” ? Figure 3 visualizes the distribution of the macro-, micro-, total porosity and bulk density analyzed for each sample and compare them between the two soil types mound soil and habitat soil. The boxplots highlight the central tendency and variability and show the presence of outliers. Box represents the interquartile range (IQR) containing the middle 50% of the data, the horizontal line inside the box the median. The whiskers extend to the smallest and largest values within 1.5 times the interquartile range from the first quartile (Q1) and third quartile (Q3). Points outside the whiskers are outliers or extremes…

Caption of Fig 3 for example:
Fig 3. Boxplots visualizing a) macro-, micro-, total porosity and b) bulk density analyzed in composite soil samples of 54 mounds built by soil-feeding termites *Patawatermes turricola* (M) and the respective control topsoil (depth 0-10 cm) taken in a distance of 5 m next to each mound (AS).

- But see the general remark above regarding the term “Adjacent Soil.”

**Lines 206ff: Legend of Table 1 (just example) – and correspondingly for Table 2 – or Table1/2 combined:**
Table 1. Means ± Standard error of physical and chemical parameters of termite-modified mound soil and recently unmodified control soil (0-10 cm) from pastures in the Colombian Amazon (n = 54). Different letters within the same row indicate significant differences at 5% (p > 0.05) as determined by Fisher’s LSD test.

**Or:**
Table 1. Physical and chemical soil parameters, comparison between termite-modified mound soil and recently unmodified control soil (0-10 cm) from pastures in the Colombian Amazon. Mean ± Standard error (n = 54) with the same letter in the same row are not significantly different at 5% (p > 0.05) as determined by Fisher’s LSD test.

→ Parameters don’t need to be listed in the legend if not abbreviated/identically named in the table

→ In case Table 1 and Table 2 will be combined, add the chemical soil parameters to table legend.

**Suggestion:** Chemical soil parameters in the table/figure could be simplified a bit, especially:
EA: exchangeable acidity, Al: exchangeable aluminum, OC: organic carbon, P: plant-available phosphorus, Ca: calcium cation, Mg: magnesium cations…

**DISCUSSION:**

**Lines 228ff:** “The findings reveal an improvement in the soil physicochemical characteristics of Mound Soil, highlighting the importance of termite activity to mediated processes in the ecosystem and its potential role in management and conservation efforts, particularly in tropical pasture landscapes.”

**Some general questions (examples)**:

Are all of the physicochemical soil parameters in mound soil improved compared to the initial habitat soil? Define "improvement" in context: beneficial e.g., for plant growth, other macrofauna, soil structure?

How can the findings on soil-feeding termite mounds of *P. turricola* be useful for habitat management or conservation efforts? Promote biodiversity by encouraging termite activity to create heterogeneous soil environments? Utilize termite mound soil as soil amendment to enhance soil fertility in managed pastures? Protect termite populations as keystone species in soil health maintenance? Use termite mounds as indicators of soil health and ecosystem integrity?

In what ways are termite mounds especially beneficial for the fertility of the habitat soil in your study region/sites? Which of the soil modifications observed may be especially beneficial for the pastures in the study region?

How do you judge the universal applicability and the quality of the results? (for study region/pastures in region)

The authors should also briefly discuss potential shortcomings of their study—for example (examples are not implying that this information is missing in the present study): Lack of data on how frequently mounds are renewed or eroded and its impact on long-term soil modification, information on soil parameters across the soil profile to discuss soil modification relative to the soil horizon from which mound soil was taken.

**Lines 239-243: Statements**

1. Mounds have lower compaction due to higher macroporosity.
2. This higher macroporosity is associated with lower microporosity.
3. which could be related to mound porosity because it is mainly constituted by macropores.
4. This is a result of termite feeding activity, which generates an increase in soil porosity/macro-pores.

Questions:

🡪 What is difference between statement 1 and statement 3?

🡪 How does soil porosity following feeding activity influence the macroporosity of mounds?

**Lines 239-243: Discussion of macro/microporosity of termite mounds in present study and comparisons with literature - example:** Authors should check if the terms (here: macro-/ microporosity) as they are used in the present study is comparable to the cited publications. In studies on termites as ecosystem engineers, macroporosity often refer to the effect of subterranean galleries and/or foraging holes in habitat soil (tunneling during feeding and nesting activities).

Reference [41]: No information regarding the macroporosity of termite mounds; instead, it discusses the porosity of habitat soil in different land uses.

Reference [42]: No information regarding the microporosity of termite mounds; it covers the porosity of surface and subsurface horizons.

Reference [43]: As far as I could see (unfortunately, no full access), no information on macropores of mound soil. This study, conducted in semi-arid Burkina Faso, assesses the use of mound soil as construction material (which termite species/feeding group?)

Reference [44] No information on macropores of termite mounds or habitat soil following feeding activities.

Reference [45] Conducted in semi-arid Burkina Faso, this study examines the effect of foraging activity and soil sheeting by fungus-growing termites on soil properties. The extensive network of subterranean galleries and foraging holes increased rainwater infiltration.

Note: Reference [45] is appropriate for Line 243 (effect of feeding activity) but does not fit its use in Line 245 (macroporosity in termite mounds).

**In general:**
Other studies often focus on different soil parameters, different feeding groups of termites, different habitat types, different soil types, or different climates. Possible deviations of findings from the present study resulting from these differences should be briefly discussed.

**Line 263:** Reference [51] studies effect of fungus-grower foraging activity 🡪 not only different feeding-type as discussed but also different biogenic structures in focus.

**Line 319f:** “These changes are likely attributed to the transfer of soil for biogenic structure construction, termite feeding activity,..”

…transfer of soil for biogenic structure construction through digestive system?

**Useful References e.g.:** (Castro Torres, 2021; Tilahun *et al.*, 2021; Franzluebbers, 2022; Duran-Bautista *et al.*, 2023)

**References in comments:**

Bates D, Maechler, M., Bolker, B., Walker, S., 2015. Fitting Linear Mixed-Effects Models Using lme4. Journal of Statistical Software 67, 1-48.

Castro Torres, J., 2021. Functional structure of termite assemblages associated with productive rubber crop systems in the northwestern colombian Amazon. Facultad de Ciencias Agrarias. Universidad Nacional de Colombia.

Donovan, S.E., Eggleton, P., Bignell, D.E., 2001. Gut content analysis and a new feeding group classification of termites. Ecol Entomol 26, 356–366.

Dray, S., Dufour, A.-B., 2007. The ade4 Package: Implementing the Duality Diagram for Ecologists. Journal of Statistical Software 22.

Duran-Bautista, E., Suárez Salazar, J., Yalanda-Sepulveda, K., 2023. Spatial distribution patterns and structure of epigeal nests of soldierless termites in livestock systems in the northwestern Colombian Amazon.

Franzluebbers, A.J., 2022. Soil mean-weight diameter and stability index under contrasting tillage systems for cotton production in North Carolina. Soil Sci Soc Am J 86, 1327-1337.

Hothorn, T., Bretz, F., Westfall, P., Heiberger, R.M., 2008. multcomp: Simultaneous Inference in General Parametric Models. Biomedical Journal 50, 346-363.

Sarcinelli, T., Schaefer, C., Fernandes-Filho, E., Mafia, R., Neri, A., 2013. Soil modification by termites in a sandy-soil vegetation in the Brazilian Atlantic rain forest. J Trop Ecol 29, 439-448.

Tilahun, A., Cornelis, W., Sleutel, S., Nigussie, A., Dume, B., Van Ranst, E., 2021. The Potential of Termite Mound Spreading for Soil Fertility Management under Low Input Subsistence Agriculture. 11, 1002.
