# Supplementary material for: Soil-feeding termites build islands of soil physical and chemical fertility in pastures in Colombian Amazon
Source: PLoS One. 2025 Apr 29;20(4):e0322186. doi: 10.1371/journal.pone.0322186 (PMC12040218; doi:10.1371/journal.pone.0322186)
Supplement: Attachment — Submitted filename: Response to Reviewer 3.docx [file pone.0322186.s004.docx]

Response to Reviewers’ Comments on PONE-D-24-16884

Ms. Ref. No.: PONE-D-24-16884_R1

Soil-feeding termites build islands of soil physical and chemical fertility in pastures in Colombian Amazon

PLOS ONE

Attention:

Kamlesh Jangid, PhD

Academic Editor, PLoS One

PLOS ONE

Dear Dr Kamlesh Jangid

Many thanks for forwarding to us the comments from the reviewer. We found the comments of the reviewer to be objective, constructive and helpful, and we have done our best to employ them wherever possible for the improvement of the manuscript. The authors have declared that no competing interests exist.

We describe below our response to comments made by reviewers. Standard font is used for the comments, while red font is used for our response to comments.

Specific responses to the Editorial Board Member and reviewers’ concerns: Our responses are in red font.

**Reviewer 3**

1. While the English grammar of your ms is almost perfect, the whole ms is still lacking of good sentence and paragraph construction. For example,

a) all paragraphs of the Intro and Discussion are very small. To fix this, I suggest to build paragraphs in these Sections of minimum 5-6 sentences.

Reply: All paragraphs now meet or exceed the 5-6 sentence requirement. They are well-developed and provide sufficient context and information.

b) Ideas, i.e., the idea of termite as modifiers of soil properties) are in all Intro paragraphs. Just use one paragraph to develop one idea. Furthermore.

Reply: We have made the suggested adjustments as shown below.

Paragraph 1: Focuses on the general role of termites as ecosystem engineers and their global significance.

Paragraph 2: Specifically addresses soil-feeding termites, their unique feeding habits, and the characteristics of their mounds.

Paragraph 3: Discusses the variability of termite mound properties and the factors influencing them.

Paragraph 4: Connects termite activity to the Amazon basin and the issue of pasture degradation.

Paragraph 5: Highlights the knowledge gap regarding *P. turricola* mounds and addresses farmer perceptions.

Paragraph 6: Presents the study's hypothesis and objectives, clearly stating the research aims.

c) Avoid repetition, for example, no need to say 6 times in the Abstract the word "unmodified".

Reply: Thank you for your feedback regarding the repetition of "unmodified" in the abstract. We have intentionally used synonyms such as "surrounding topsoil" and "adjacent soil" throughout the document to reduce redundancy.

d) No need to build a whole subsection on termite identification (Line 167) when you are dealing with only 1 species. Similarly, no need for the last section of Conclusions (just tight ideas in the Discussion)

Reply: In response to your observation regarding the "Termite identification" and "Conclusions" subsections, we have removed these subsections and integrated the relevant information into the appropriate sections of the manuscript, as you suggested.

e) Use terminology constant. The ideas of fertility *Islands* are only use in the title, in one sentence in the Intro, and in Discussion. Incorporate this concept in the Design, statistical framework, Images and tables. Remember, the main objective of your study was to test **that mound-building activities by soil-feeding termites improve the soil physical and chemical properties by creating island of soil fertility**. You need to show this in your ms.

Reply: We have reinforced the idea of termite-built “fertility islands” throughout the manuscript. To ensure consistency and emphasize our main objective, we have incorporated this concept in several key areas

Table and Figure Titles

Study area and sampling design: line 91, 99 to 100 and 110

Data analysis: lines 165 to 166 and 181 to 182

f) Be humble. in Line 84-84 you hypothesize that fertility islands **can be particularly important in those nutrient-poor agroecosystems**. While this is a nice idea, you are not testing it in this ms. So, just don't build sentences you are not mean to test. Leave speculation for the Discussion.

Reply: Thank you for your feedback. In response to your suggestion, we have removed the statement about the particular importance of fertility islands in nutrient-poor agroecosystems from lines 84-84.

2. The previous reviewers gave you many ideas (i.e., building structures from surrounding organic materials not necessarily increase fertility, just redistribute resources? Please pay more attention to their comments and incorporate them into the text as alternative hypothesis (or falsify them with your data). Please recognize the limitations of your study. Doing this only makes your study stronger.

Reply: We appreciate the valuable ideas provided by the previous reviewers. We have addressed their comments. Specifically, we have addressed the possibility that constructing structures from surrounding organic materials represents a redistribution of resources rather than a net increase in fertility. Furthermore, we have acknowledged and discussed the limitations of our study, as suggested (Lines 273 to 282, 304 to 308, 363 to 373, 375 to 383).

3. Be more careful with the presentation of your work. I could not find figure 3.

Reply: We sincerely apologize for the inconsistency in the submission regarding Figure 3. We assure you that this oversight will be corrected in the revised version.
